# Alcohol Seeking Under Risk of Punishment Is Associated With Activation of Cortical and Subcortical Brain Regions

**DOI:** 10.3389/fnbeh.2021.739681

**Published:** 2021-10-21

**Authors:** Allison J. McDonald, Isis Alonso-Lozares, Vasco Rauh, Yvar van Mourik, Dustin Schetters, Taco J. De Vries, Nathan J. Marchant

**Affiliations:** Department of Anatomy and Neurosciences, Amsterdam Neuroscience, Amsterdam UMC, Vrije Universiteit Amsterdam, Amsterdam, Netherlands

**Keywords:** alcohol, addiction, relapse, Fos, conflict

## Abstract

In humans, stimuli associated with alcohol availability can provoke relapse during abstinence. In this study, we investigated the role of discriminative stimuli (DS) in the control of alcohol seeking in two types of behavioral tests. The first test examined the ability of an alcohol-associated DS to promote alcohol seeking (relapse) after punishment-imposed abstinence in the presence of a different DS. Following this, we tested whether the differentially associated DS can promote and suppress alcohol self-administration in a within-session discrimination task. During the within-session discrimination task, we also tested the rate of alcohol self-administration when two DS are presented in a compound. We first trained Long-Evans male rats (*n* = 24) to self-administer alcohol in the presence of one DS (reward-associated discriminative stimulus, rewDS) and then punished that behavior in the presence of a different DS (punishment-associated discriminative stimulus, punDS). On the test, we found that rats tested with the rewDS showed higher alcohol seeking than rats tested with the punDS. This result shows that a single Cue DS can promote alcohol seeking in a manner comparable to contexts. Subsequently, we trained 16 of these rats in a within-session trial-based discrimination task, comprised of intervening 2-min trials of rewDS, punDS, or conflict with rewDS and punDS in compound and a reduced probability of punishment. We found that alcohol self-administration is bi-directionally regulated by the rewDS and punDS. In conflict trials, alcohol self-administration was at a rate that was intermediate between the rewDS and punDS trials. In a final test, rats were presented with one of the three trial conditions and perfused for Fos immunohistochemistry. We found Fos expression was higher in the rats tested in the conflict condition in three interconnected sub-cortical brain regions. This study demonstrated the important role that alcohol-associated DS plays an important role in promoting relapse to alcohol seeking after punishment-imposed abstinence. We also implemented a within-session discrimination task that allows for the study of alcohol seeking under motivational conflict, which may be relevant for alcohol use despite negative consequences. The results from the Fos data suggest that higher alcohol seeking in approach-avoidance motivational conflict is associated with activation of sub-cortical regions but not cortical regions.

## Introduction

In humans, places or contexts previously associated with alcohol use can provoke relapse during abstinence (Wikler, 1973; O’Brien et al., 1992). We have previously studied this phenomenon using the context-induced relapse model, where alcohol taking is punished in an alternative context (Marchant et al., 2013). The introduction of punishment incorporates a critical component of alcohol addiction, a voluntary motivation to abstain from alcohol use out of a desire to avoid the negative consequences (Klingemann, 1991; Blume et al., 2006; Hasin et al., 2013; Marchant et al., 2019). We and others have shown that punishment, similar to extinction, is encoded as a distinct context-dependent memory that does not impact original associations (Marchant et al., 2013; Bouton and Schepers, 2015).

In studies of relapse, two different environmental contexts are used to signal whether a response will be reinforced with alcohol or in the alternative context with either extinction or punishment (Hamlin et al., 2007; Marinelli et al., 2009; Marchant et al., 2013). These environmental stimuli retain the ability to trigger relapse through associative conditioning that occurs during initial learning (Bouton et al., 2020). The associative mechanism by which contexts promote drug seeking after extinction is thought to be comparable to an occasion setter (Crombag et al., 2008), which is a type of stimulus that defines whether another stimulus or an operant response will be reinforced (Holland and Bouton, 1999; Fraser and Holland, 2019). Discriminative stimuli (DS), in discriminated operant paradigms, are also thought to act in a manner that is comparable to occasion setters. Drug relapse studies have previously shown that a DS associated with drug availability can reinstate alcohol seeking after extinction in the absence of any DS (Katner et al., 1999; Cannella et al., 2009). While we have previously shown that an alcohol-associated context can reinstate alcohol seeking after punishment-imposed abstinence in a different context (Marchant et al., 2013), to date, no study has tested whether an alcohol-associated DS can reinstate alcohol seeking after punishment-imposed abstinence in the presence of another DS.

While relapse is a critical factor in the treatment of alcohol addiction, one of the core criteria that characterizes addiction is compulsive use (Everitt et al., 2008; Naqvi et al., 2014). Compulsive or aversion-resistant (Hopf and Lesscher, 2014), alcohol use is defined as continued drug use despite the knowledge of the negative consequences that occur because of drug use, such as loss of job, relationship breakdowns, and drug use in the face of danger (Hasin et al., 2013). One advantage that a single Cue DS has over a context is that these associations can be tested in within-session in different trials to determine how well they are able to exert control of alcohol seeking and taking. Within-session discrimination tasks are common (Taha and Fields, 2006; Ambroggi et al., 2011), but there are few studies that test discrimination of alcohol and punishment of alcohol self-administration in rats. The use of a single stimulus (rather than a context) as a DS signaling reward or punishment provides a novel opportunity to place two previously learned stimuli in conflict by presenting them in a compound. Because the two stimuli signal that the same response (lever press) will cause motivationally opposing outcomes, such a test induces a state of approach-avoidance motivational conflict (Miller, 1944; Gray and McNaughton, 2000; McNally, 2021) which can be identified by oscillating behavioral response or omission and increased response latency. This psychological construct perhaps best characterizes the conflicting nature of competing motivations that are present in alcohol use despite negative consequences (Hopf and Lesscher, 2014).

In this study, we aimed to test whether a DS associated with alcohol self-administration can promote relapse of alcohol seeking after punishment-imposed abstinence in the presence of a different DS. Following this, we aimed to test whether these two DS can control alcohol self-administration on a shorter time scale, using a within-session task that presents each of the DS for 2 min over successive trials. In addition, we tested the effect of presenting these DS in compound (conflict) on the rate of alcohol seeking. This is a novel approach to assess the behavioral and neurobiological mechanisms of alcohol seeking in the face of negative outcomes. Finally, we used Fos as a marker of neuronal activity to identify brain regions associated with the DS control of alcohol seeking in either reward, punishment, or conflict conditions.

## Materials and Methods

### Subjects

We obtained 24 male Long-Evans rats, aged 12–16 weeks upon arrival, from Janvier Labs (France). In compliance with Dutch law and Institutional regulations, all animal procedures were approved by the Centrale Commissie Dierproeven (CCD) and conducted in accordance with the Experiments on Animal Act. Experiments were approved by the local animal welfare body Animal Experiments Committee of the Vrije Universiteit, Amsterdam, Netherlands. Behavioral tests were conducted during the dark phase of the diurnal cycle of rat (12 h/12 h). Food and water were available *ad libitum*. We pair-housed the rats throughout the experiment.

### Apparatus

All data were collected through the MED-PC IV program (Med Associates, Georgia, VT, United States). Each chamber had two retractable levers on one wall. The left lever was designated “active,” and the right lever was designated “inactive.” Between the two levers, there was a receptacle magazine connected to a syringe pump for alcohol delivery, which had an infrared beam to measure head entries. Above the active lever, there was a light panel with three small lights (red, green, and yellow). On the opposite side of the chamber, there was a white house light and a white-noise generator. The grid floor was connected to shock controllers.

### Behavioral Procedure

#### Phase 1: Intermittent Access to Alcohol in the Home-Cage

We used an intermittent access (3–4 times/week) alcohol procedure (Wise, 1973; Simms et al., 2008) in which rats received 12 × 24 h sessions of access to one bottle of 20% alcohol and one water bottle. We prepared alcohol solutions in tap water from 100% (v/v) ethanol in standard rat water bottles. Daily sessions began at 09:00 a.m. After 24 h, we replaced the alcohol bottle with a second water bottle for the subsequent 24–48 h alcohol-free days. The following day, the second water bottle was replaced with the 20% alcohol bottle, and the location of the alcohol bottle was alternated from the previous session. Total alcohol consumption in grams was calculated for each session, using the weight difference between the beginning and end of the session, minus 2 g for spillage, multiplied by 0.97 (density of 20% ethanol).

#### Phase 2: Alcohol Self-Administration

Alcohol self-administration sessions lasted 30 min. One to two min after placing the rat in the chamber, the session started with insertion of the levers into the chamber, and switching on the DS (reward-associated discriminative stimulus, rewDS; house-light or white-noise, counterbalanced). Responses on the active lever were reinforced with an infusion of 0.2 ml of 20% alcohol into the magazine. Reinforced lever presses resulted in the presentation of a conditioned stimulus (CS) comprised of three lights above the lever, which were illuminated for 10 s. During this time, responses were recorded but had no consequence (10 s time out). Responses on the inactive lever had no consequence throughout. We first trained the rats on a fixed-ratio (FR)-1 schedule for six sessions, which was then increased to FR-2 for the remaining sessions.

#### Phase 3: Punishment of Alcohol Self-Administration

We gave punishment sessions in the same operant chamber as alcohol self-administration. One to two min after placing the rat in the operant chamber, the session started with insertion of the levers and switching on the opposite DS (punishment-associated discriminative stimulus, punDS; house-light or white-noise, counterbalanced). Punishment sessions lasted 30 min. The reinforcement schedule for alcohol was FR-2, and 100% of the alcohol-reinforced active lever presses resulted in foot shock. The intensity was 0.25 mA for the first three sessions and 0.30 mA for the final, i.e., the fourth session.

#### Phase 4: Test for Discriminative Cue-Induced Reinstatement of Alcohol Seeking

The rats were tested in extinction conditions without alcohol deliveries or shock. Responses on the active lever resulted in the CS being turned on for 10 s, on an FR-2 schedule of reinforcement. We tested half of the rats under the reward DS and the other half under the punishment DS.

#### Phase 5: Within-Session Discriminative Control of Alcohol Self-Administration

We further trained 16 of the rats from the previous phases in the within-session DS sessions. Each session was comprised of 20 trials of 2-min duration, separated by 1 min of inter-trial-interval (ITI). There were three types of trials: reward, punishment, and conflict. In the reward trials, the rewDS was turned on and during this time active lever presses resulted in alcohol delivery on an FR-2 schedule of reinforcement. In the punishment trials, the punDS was turned on and during this time active lever presses (FR-2) resulted in both alcohol delivery but 100% of the alcohol-reinforced active lever presses also resulted in foot-shock. In the conflict trials, both the rewDS and punDS were presented in a compound, and during this time, active lever presses (FR-2) resulted in both alcohol delivery but the probability of punishment on a reinforced active lever press was 50%. During the ITI, the levers were retracted from the chamber, and the DS were turned off. The order was an alternating design, whereby the rewDS was always first, and next was conflict, followed by rewDS, punDS, and then rewDS, and this pattern repeated. In the first session, there were 12 rewards, three punishments, and five conflict trials when the probability of shock was only 25%. For all subsequent sessions, there were 10 reward trials, five punishment trials, and five conflict trials. In sessions 1–8, the shock intensity was 0.25 mA, and in sessions 9–12, the shock intensity was increased to 0.3 mA.

#### Phase 6: Test Session and Perfusion for Fos Immunohistochemistry

On the final test day, the rats were given the same DS for 10 × 2-min trials with a 1-min ITI. Five rats received 10 × rewDS trials, five rats received 10 × punDS trials, and six rats received 10 × conflict trials. During this test, the reinforcement schedule was identical to the previous phases, that is, responses on the active lever resulted in alcohol in the rewDS conditions, 100% foot shock punishment in the punDS condition, and 50% foot shock punishment in conflict. The shock intensity was 0.3 mA for punDS and conflict. Sixty min after the end of the session, the rats were taken from the chamber and perfused.

### Immunohistochemistry

Following the final test session, we deeply anesthetized the rats with isoflurane and Euthasol^®^ injection (i.p.) and transcardially perfused them with ∼50 ml of normal saline followed by ∼400 ml of 4% paraformaldehyde in 0.1 M sodium phosphate (pH 7.4). The brains were removed and post-fixed for 2 h and then put in 30% sucrose in 0.1 M phosphate-buffered saline (PBS) for 48 h at 4°C. The brains were then frozen on dry ice, and coronal sections were cut (40 μm) using a Leica Microsystems cryostat and stored in 0.1 M PBS containing 0.1% sodium azide at 4°C.

We selected a 1-in-4 series of sections from each rat and used immunofluorescence to label Fos positive neurons. We washed free-floating sections (3 × 10 min) in PBS. We generated a trial reaction by adding PBS containing 0.5% TritonX-100 (PBS-Tx) with 10% normal donkey serum (NDS), which was incubated for 2 h. Next, we incubated the sections for 48 h at 4°C in PBS-Tx with 2% NDS, rabbit anti-c-Fos primary antibody (1:2,000; Cell Signaling #5348). After washing off unbound primary antibodies, sections were incubated for 2 h in PBS-Tx with 2% NDS and donkey anti-rabbit AF-594 secondary antibody (1:500; Molecular Probes #A21207). We then rinsed sections in PBS and added PBS containing 4′,6-diamidino-2-phenylindole (DAPI; 1:5,000) for 10 min. After another round of washes, we mounted the sections onto gelatin-coated glass slides, air-dried, and cover slipped with Mowiol containing 2.5% 1, 4-Diazabicyclo-octane (DABCO).

### Image Acquisition and Neuronal Quantification

We digitally captured images of immunoreactive cells with a 10x objective using a Vectra Polaris slidescanner. We identified Fos-labeled neurons using the CY3 filter (exposure: 80 ms) and DAPI-labeled neurons using the DAPI filter (exposure: 1 ms). We analyzed sections in the following bregma coordinates: Bregma +3.72 mm, Bregma +1.44 mm, and Bregma −2.76 mm. The brain regions were defined according to the fifth edition of the Paxinos brain atlas (Paxinos and Watson, 2005). We performed quantification using the cell detection feature in QuPath (Bankhead et al., 2017), applying a constant set of parameters throughout each brain region/rat. We present our data as the total number of identified Fos neurons divided by the area of the analyzed region in mm. The analyzed regions are: Bregma +3.2 mm: Cg, cingulate cortex; dmPFC, dorsomedial prefrontal cortex; vmPFC, ventromedial prefrontal cortex; LO, lateral orbitofrontal cortex; VO, ventral orbitofrontal cortex; RAIC, rostral agranular insular cortex. Bregma +1.5: MAIC, mid agranular insular cortex; Core, Nucleus Accumbens Core; Shell, nucleus accumbens shell; DLS, dorsolateral striatum; DMS, dorsomedial striatum; LS, lateral septum. Bregma −2.5 mm: PVT, paraventricular nucleus of the thalamus; BLA, basolateral amygdala; CeA, central nucleus of the amygdala; LH, lateral hypothalamus; PAIC, posterior agranular insular cortex.

### Statistics

We performed all statistical analyses using IBM SPSS V21. For the first four phases (relapse experiment) we analyzed the behavioral data separately for the different phases. The dependent variables for all phases were the total number of active and inactive lever presses. We also used the alcohol reward deliveries as dependent variables for the alcohol self-administration and punishment phases. We used repeated measures ANOVA to test for the main effect of session for alcohol self-administration (rewDS) and punishment (punDS), using the within-subjects factor lever (Inactive and Active). For the relapse tests, we analyzed the data using repeated measures ANOVA with between-subjects factor Test Cue (rewDS and punDS) and the within-subjects factor lever (Active and Inactive). For the latency measures, we used the between-subjects factor Test Cue (rewDS and punDS) in an independent samples *t*-test.

For the within-session DS phase of the experiment, to make comparisons between different numbers of trials we divided the total lever presses (active and inactive) by the total amount of time (in min) that was given for each Cue type to produce a comparable rate. No responses were made during the ITI because the levers were retracted during this period. We used repeated measures ANOVA to test for the main effect of session using the within-subjects factor lever (Inactive and Active), and Test Cue (rewDS, punDS, and conflict). The suppression ratio (SR) was calculated based on the rate of responding on the active lever in punDS, or conflict is compared to the rate of responding on the active lever in rewDS in that session (e.g., SR = punDS/punDS + rewDS). We used Pearson correlation to assess the relationship between the average rate of responding in the final four sessions for conflict and punDS trials, and for the average rate of responding in a rewDS trial, following either punDS or conflict trials, and for the overall average rate of responding in rewDS, punDS, and conflict trials. We analyzed the Fos data in each brain region separately using a one-way ANOVA to test an effect of Test Cue (rewDS, punDS, and conflict), follow-up *post-hoc* tests were conducted (Fisher’s Least Significant Different) on regions that have the significant main effect of Test Cue.

## Results

### Discriminative Stimuli-Induced Relapse of Alcohol Seeking After Punishment-Imposed Abstinence

**FIGURE 1:**
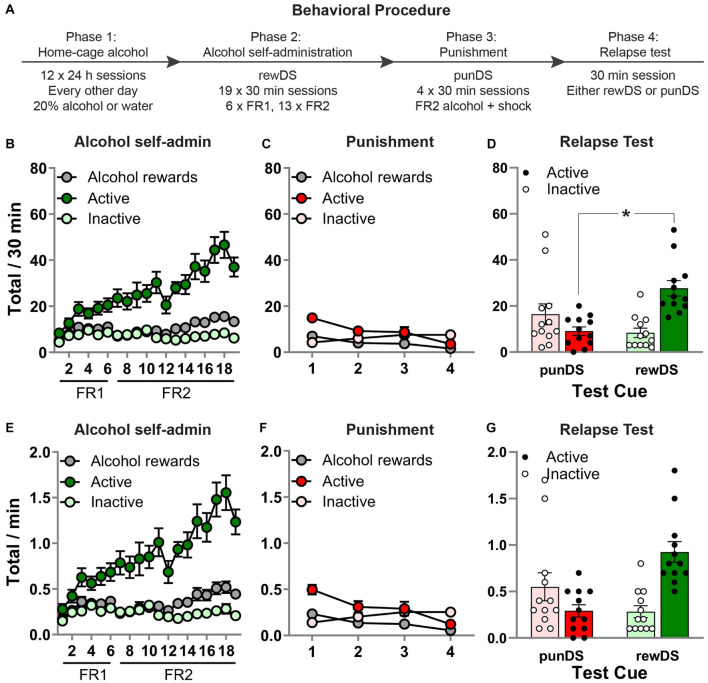
Discriminative stimulus-induced relapse after punishment-imposed abstinence. **(A)** Outline of the experimental procedure. **(B)** Mean ± SEM number of active and inactive lever presses and alcohol rewards, during alcohol self-administration in the presence of the alcohol-associated DS (rewDS). **(C)** Mean ± SEM number of active and inactive lever presses and alcohol rewards, during punishment of alcohol self-administration in the presence of the punishment-associated DS (punDS). **(D)** Mean ± SEM number of active and inactive lever presses during the relapse test. **(E–G)** These show the same data expressed as rate of responding per 1 min. *p < 0.05, punDS *n* = 12; rewDS *n* = 12. FR, fixed-ratio. punDS, punishment-associated discriminative stimulus; rewDS, reward-associated discriminative stimulus.

Figure 1A shows the experimental outline of the relapse phase of the experiment. During the home-cage alcohol access phase (data not shown) the rats consumed approximately 4.5 (±0.98) g/kg/24 h on day 1 and 7.1 (±1.62) on day 12. Despite this increase, we did not observe a significant effect of session on g/kg/24 h intake [*F*_(11,121)_ = 1.2, *p* > 0.05].

Figure 1B shows behavior during the alcohol self-administration phase. All rats acquired alcohol self-administration in the presence of the rewDS, as indicated by a significant increase in responses on the active lever compared to the inactive lever [session × lever interaction: *F*_(18,414)_ = 9.5, *p* < 0.05]. There was also a significant increase in alcohol rewards throughout the sessions [*F*_(18,414)_ = 5.9, *p* < 0.05]. During the final three sessions, the total amount of alcohol consumed was 0.80 (±0.10) g/Kg, which may potentially be equivalent to blood ethanol concentration (BEC) of approximately 40 mg/dl. However, we did not measure BEC, and Long-Evans rats have been shown to have a very steep BEC to g/Kg relationship (Simms et al., 2008). Figure 1C shows behavior during the punishment phase in the presence of the punDS. Alcohol self-administration was significantly decreased by the introduction of response-contingent foot-shock punishment. Across the four sessions, there was a significant decrease in responses on the active lever [*F*_(3,69)_ = 11.9, *p* < 0.05] and alcohol deliveries [*F*_(3,69)_ = 15.9, *p* < 0.05]. Interestingly, there was a significant increase in responses on the inactive lever [*F*_(3,69)_ = 4.9, *p* < 0.05].

Figure 1D shows behavior in the test session. We found that rats tested with the rewDS had significantly higher alcohol seeking compared to rats tested with the punDS. There was a significant Test Cue × lever interaction [*F*_(1,22)_ = 21.6, *p* < 0.05]. We also found a significant effect on latency to the first active lever press [rewDS = 58.1 (±11.4) s, punDS = 476.5 (±191.8) s; *t*_(22)_ = −2.2, *p* < 0.05]. These data show that DS can act in a similar manner to contexts, causing reinstatement of alcohol seeking after punishment-imposed abstinence. Figures 1E–G show the same data presented as totals per minute, for comparison to the subsequent phases of the experiment.

### Within-Session Discriminated Alcohol Self-Administration, Punishment, and Conflict

**FIGURE 2:**
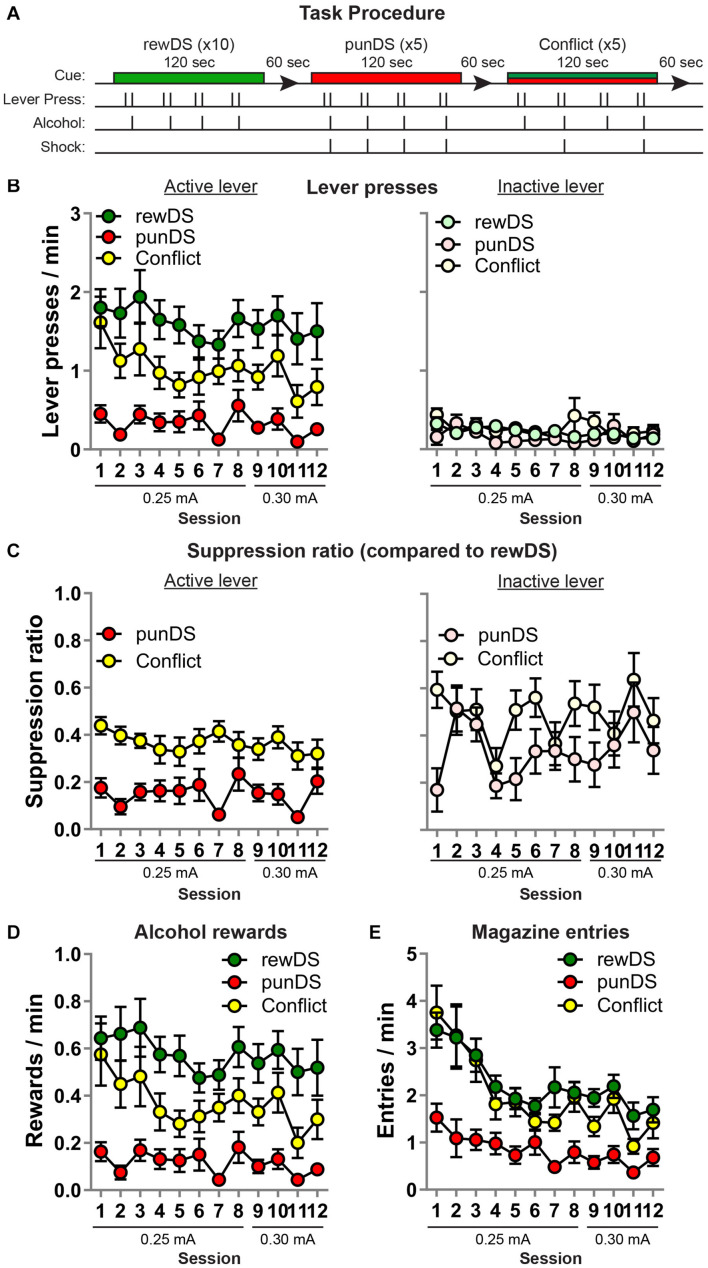
Within-session discriminated alcohol self-administration and punishment (*n* = 16). **(A)** Outline of task procedure. During rewDS trials, active lever presses resulted in alcohol reward delivery on an FR-2 schedule. During punDS trials, active lever presses resulted in alcohol reward delivery on an FR-2 schedule, and 100% of the reinforced responses were punished. During conflict trials, the probability of punishment was 50%. **(B)** Mean ± SEM rate of active (left) and inactive (right) lever presses during the sessions. **(C)** Mean ± SEM suppression ratio of active (left) and inactive (right) lever presses in punDS and conflict trials relative to the rate during rewDS trials. **(D)** Mean ± SEM rate of alcohol rewards during the sessions. **(E)** Mean ± SEM rate of entries into the alcohol receptacle during the sessions.

After the relapse test, we trained the rats in a within-session DS task. Figure 2A shows the task design for a single session (there were 12 sessions in total). Overall, the amount of alcohol consumed in these sessions was comparable to that observed in alcohol self-administration in Phase 2 (0.74 g/Kg ± 0.11; data not shown). We found that alcohol self-administration is significantly higher in the presence of the rewDS compared to the punDS and in compound (conflict), and in the conflict trials, alcohol self-administration was significantly higher than during the punDS trials (Figure 2B). Overall analysis revealed the main effect of Cue [*F*_(2,30)_ = 35.2; *p* < 0.05] with no Cue × session interaction [*F*_(22,330)_ = 0.8, *p* > 0.05]. Alcohol self-administration in the rewDS trials was stable across the 12 sessions [*F*_(11,165)_ = 0.65, *p* < 0.05]. A similar pattern of statistical results was found on analyses of the alcohol rewards (Figure 2D). To compare the magnitude of suppression of alcohol self-administration in punDS and conflict trials, we calculated a SR of the rate of active lever presses in punDS and conflict compared to the rewDS rate (Figure 2C, left). Using the SR, we found a significant effect of Cue [*F*_(1,15)_ = 41.3, *p* < 0.05], and no Cue × session interaction [*F*_(11,164)_ = 1.8, *p* > 0.05]. Thus, the rats showed significantly greater suppression of alcohol self-administration in the presence of the punDS compared to in conflict.

On inactive lever presses (Figure 2B, right), the effect of Cue on the rate of responding approached significance [*F*_(2,30)_ = 2.9, *p* = 0.07], suggesting that the effects of punishment are specific to the active lever. However, using the SR data (Figure 2C, right), we found a significant effect of Cue [*F*_(1,15)_ = 8.4, *p* < 0.05] and no Cue × session interaction [*F*_(11,165)_ = 1.4, *p* < 0.05]. This suggests that the inactive lever presses are also suppressed in punDS trials compared to conflict, at least relative to the rewDS trials.

On the head entries into the alcohol magazine (Figure 2E), we found a main effect of Cue [*F*_(2,30)_ = 49, *p* < 0.05] and a session × Cue interaction [*F*_(22,330)_ = 2.2, *p* < 0.05], likely reflected by the higher magazine entries in rewDS and conflict trials in the earlier sessions compared to the later sessions.

**FIGURE 3:**
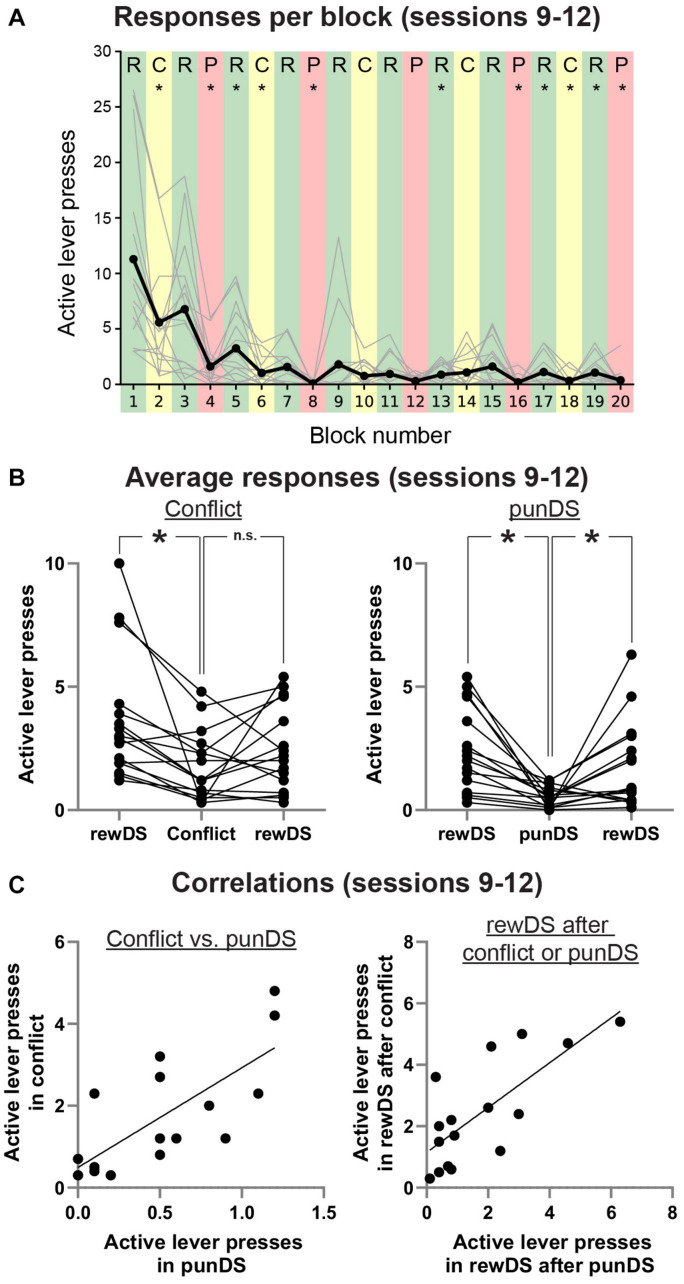
Bi-directional regulation of alcohol self-administration by the reward and punishment associated DS. **(A)** Average (dark line) and individual (gray lines) active lever presses within each trial type (Green: rewDS, yellow: conflict, red: punDS), over the final four sessions when both the trial order and shock intensity were the same (0.3 mA). **(B)** Individual data of the average responses in these sessions during consecutive rewDS, conflict, rewDS trials (Left) and consecutive rewDS, punDS, rewDS trials (right). **(C)** Correlations in the individual rat mean active lever presses during conflict and punDS trials (Left) and in the rewDS trials following either a conflict or punDS trial (right). *p < 0.05.

As a final test of within-session discrimination between the different DS conditions, we averaged the total active lever presses from each trial in the final four sessions, when the shock intensity was set at 0.3 mA. Figure 3A shows the mean active lever presses for all rats, with individual data also presented. We used repeated measures *t*-tests to compare responses in each trial to the trial preceding it. We found that four of the five punDS trials were significantly lower than the preceding rewDS session, and three of the five of the conflict trials were significantly lower than the preceding rewDS session. Of the rewDS trials following either punDS or conflict (nine in total), four were significantly higher. In Figure 3B, we show the average active lever presses in conflict trials (Left) and in punDS trials (right), and the average of the rewDS trials preceding and following. Overall, responding in conflict was significantly lower than the rewDS trial preceding it, but did not significantly increase in the trial after. For punDS, responding was significantly decreased compared to the preceding rewDS trial and significantly increased in the subsequent rewDS trial. These data show that within-session responding was bi-directionally controlled by the associations of the punDS and rewDS. For conflict, the control is less clear, but this is partly explained by the variability that is observed in response-contingent punishment when the probability of shock is not 100% (Marchant et al., 2018).

In Figure 3C, we show correlations between total active lever presses in conflict and in punDS trials (Left). We found a significant correlation between these responses [*r*_(14)_ = 0.74, *p* > 0.001]. This indicates that the level of responding in conflict and punDS trials is a function of the amount of suppression that the response-contingent shock causes. We found (data not shown) no correlation between the rate of responding in rewDS and punDS [*r*_(14)_ = 0.25, *p* < 0.05] or in conflict [*r*_(14)_ = 0.45, *p* < 0.05], possibly indicating that the relationship between responding in rewDS trials is unrelated to suppression in punDS or conflict trials. Figure 3C right shows correlations between the recovery of responding in rewDS after conflict and punDS trials. This too was significant [*r*_(14)_ = 0.75, *p* > 0.001], demonstrating that the rats that were likely to engage in alcohol seeking after punDS trial also did so after conflict.

### Neural Activity (Fos) Associated With Discriminated Alcohol Self-Administration, Punishment, and Risk of Punishment in Conflict

**FIGURE 4:**
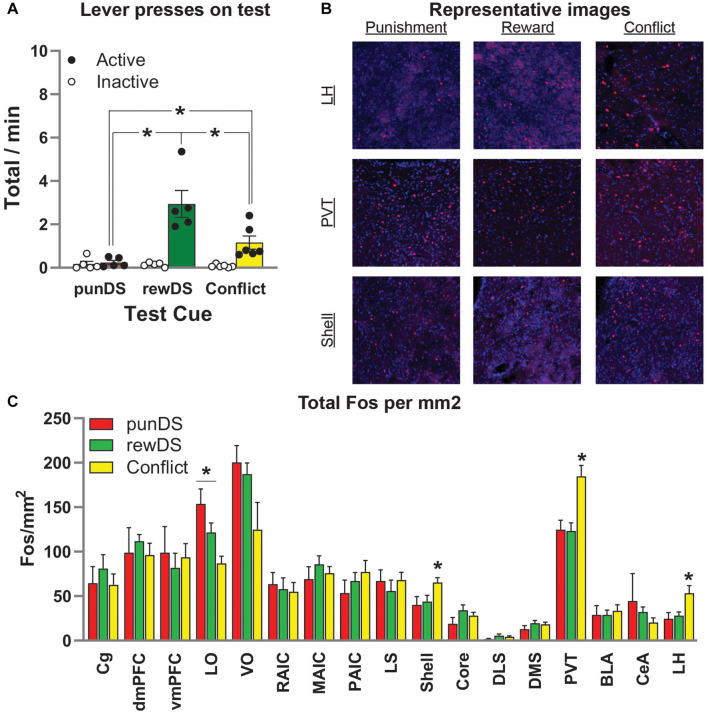
Neuronal activity (Fos) is associated with alcohol seeking under risk of punishment. **(A)** Mean ± SEM (individual data in dots) rate of active lever presses in the final reinforced test where rats received 10 × 2 min trials of either punDS (*n* = 5), rewDS (*n* = 5), or conflict (*n* = 6). **(B)** Representative images of Fos positive neurons in lateral hypothalamus (LH), paraventricular thalamus (PVT), and nucleus accumbens shell. **(C)** Fos neurons per mm^2^ in the regions of interest assessed. *p < 0.05.

Figure 4A shows the behavior from the final, reinforced, test session where rats were tested with 10 identical DS trials (either rewDS, punDS, or conflict), each lasting 2 min with a 1-min ITI. We found that responding in this session was comparable to the previous within-session discrimination sessions. There was a significant Test Cue x lever interaction [*F*_(2,13)_ = 11.2, *p* < 0.05]. Post-hoc tests on active lever presses revealed significant differences between punDS and conflict groups (*p* < 0.05), rewDS and punDS groups (*p* < 0.05), and rewDS and conflict groups (*p* < 0.05). There were no significant differences between the groups on responses on the inactive lever (*p* > 0.05). Interestingly, for the total count of magazine entries during the test (data not shown) was significantly higher for the conflict and rewDS groups compared to the punDS group (*p* < 0.05), and there was no difference between the conflict and rewDS group (*p* > 0.05). This shows that while the rewDS group received more alcohol on the final test, the amount of time spent in the magazine was not different.

Figure 4B shows representative images of Fos-labeled neurons in the LH, the PVT, and in the nucleus accumbens shell (Shell). In Figure 4C, we show the total counts of Fos per mm^2^ for all regions we assessed. ANOVA revealed a significant effect of Test Cue in the following regions: LH [*F*(2,14) = 5.1; *p* < 0.05], LO [*F*(2,14) = 8.0; *p* < 0.05], PVT [*F*(2,14) = 10.5; *p* < 0.05], and NAc Shell [*F*(2,14) = 3.8; *p* = 0.05]. Follow-up post-hoc revealed the following effects for comparison between conflict and rewDS: LH (*p* = 0.02), LO (*p* = 0.058), PVT (*p* = 0.002), and NAc Shell (*p* = 0.05); comparison between conflict and punDS: LH (*p* = 0.02), LO (*p* = 0.002), PVT (*p* = 0.003), NAc Shell (*p* = 0.03); comparison between rewDS and punDS: LH (*p* > 0.05), LO (*p* > 0.05), PVT (*p* > 0.05), and NAc Shell (*p* > 0.05). In summary, these findings show that we found no significant differences in Fos expression between the rewDS and punDS rats. In LH, PVT, and Shell, we found a significant difference between rats tested in conflict versus both rewDS and punDS. Interestingly, for LO we found that punDS had higher Fos expression compared to conflict. Whereas in LH, PVT, and Shell, we found that conflict was higher than both rewDS and punDS.

## Discussion

Here we report four main findings. The first is that an alcohol-associated DS induces alcohol seeking after punishment-imposed abstinence in the presence of a different DS. This effect is comparable to the context-induced relapse after punishment-imposed abstinence model (Marchant et al., 2013) and shows that a single stimulus can serve in a manner comparable to a context in this design. The second is that a DS associated with reward or punishment can both promote and suppress alcohol seeking in a within-session time-scale. We found that within-session control of alcohol self-administration is achieved in a task with alternating trials of rewDS and punDS. The third is that presentation of the rewDS and punDS in compound, with reduced probability of punishment, achieves a level of alcohol self-administration that is both higher than punDS and lower than rewDS. The fourth is that brain activity, as indexed by expression of Fos, associated with motivational conflict is higher in PVT, NAc Shell, and LH, while during conflict we found that Fos expression was lower in LO compared to the punDS tested rats.

### Role of Discriminative Stimuli in Relapse to Alcohol Seeking After Punishment

We show that a single stimulus (light or sound) can serve as a DS that can promote alcohol seeking in a relapse test after punishment-imposed abstinence. The test was conducted in extinction conditions, as such this procedure is very similar to our previous model of context-induced relapse after punishment-imposed abstinence (Marchant et al., 2013). This has practical relevance to rodent models of alcohol and drug relapse. We show that the DS can act in a similar manner to a context, and that a single stimulus can act in a comparable manner to a context. To our knowledge this is the first time that DS-induced reinstatement of alcohol seeking has been observed after punishment-imposed abstinence. While this study shows that a DS can act in a manner comparable to a context, another study recently found that a DS can induce relapse to cocaine seeking after abstinence (Madangopal et al., 2019, 2021). Critically, Madangopal et al. show that this relapse is potentiated in a manner comparable to incubation of drug seeking (Grimm et al., 2001), demonstrating an important difference between contextual and discriminative Cues.

Contexts have been proposed to act in a similar manner to Pavlovian “occasion setters,” which determine whether the response-alcohol association or the response-punishment association is expressed (Crombag et al., 2000, 2008). However, the original conceptions of occasion setters come from Pavlovian conditioning designs (Holland, 1992; Fraser and Holland, 2019), and recent work with instrumental conditioning designs have provided evidence that contexts do not operate as occasion setters in instrumental conditioning, rather the context becomes directly associated with the response (Todd et al., 2014). Here, by replicating the context-induced relapse after punishment effect (Marchant et al., 2013) using a single stimulus DS, we show that a DS can act in comparable manner to contexts in terms of relapse to alcohol seeking. In future studies, it will be of interest to determine if the DS becomes directly associated with the response, or if the different nature of the stimulus (i.e., context versus single stimulus) changes the associative structure underlying the behavioral responses to the different DS.

### Discriminative Stimuli and the Control of Alcohol Seeking on a Shorter Time-Scale

Discriminated punishment has been observed previously with rats responding for a food reward (Bolles et al., 1975, 1980). However, to our knowledge, this is the first time that DS have been used to discriminate between alcohol-reinforced responding and punishment of alcohol-reinforced responding. One advantage of using a single stimulus DS over contexts is that the DS can be turned on or off within-session. We used this approach to test whether alcohol seeking and punishment are DS controllable in a shorter time frame. We found that the rats are able to both decrease and increase their rate of alcohol seeking and taking in response to the differentially associated DS. While alcohol responding does decrease through the session (Figure 3A), the rate of responding was found to significantly decrease during punDS and the significantly increase in the subsequent rewDS trials.

We also show that presenting the two DS in compound, and reducing the probability of shock to 50%, is sufficient to motivate the rats to increase alcohol seeking, potentially inducing a greater state of approach-avoidance motivational conflict than in the certain punishment trials (Miller, 1944; Gray and McNaughton, 2000; Pennartz et al., 2011; Ito and Lee, 2016; McNally, 2021). Implementation of the conflict trials opens possibility to probe the neural substrates of conflicting motivations of approach and avoidance. The resolution of this conflict is an important psychological mechanism underpinning alcohol use despite negative consequences (Naqvi et al., 2014) and for other psychological disorders, such as anxiety (Gray and McNaughton, 2000) and obsessive-compulsive disorder (Figee et al., 2011; Milad et al., 2013). We found that the rate of alcohol taking in conflict is intermediate between unpunished rewDS and certain punishment of punDS. The rate of punishment in the conflict trials (50% of reinforced responses) is comparable to what we have used in the previous studies (Marchant et al., 2013, 2014, 2016).

One aspect of the conflict condition that we have not tested here is whether the critical feature is presentation of the Cues in conflict or the reduced shock probability. It is likely that the behavior of rat is sensitive to the reduced shock probability because it is well known that the probability of shock punishment impacts punishment suppression (Murray and Nevin, 1967). However, the magazine entry data (Figure 2E) provide some evidence of increased alcohol seeking beyond what is observed in the punDS trials. We found that entries into the magazine did not differ between the conflict and rewDS trials, and both were higher than in punDS. This is surprising since the rats receive less alcohol deliveries during the conflict trials compared to rewDS trials. We propose that this observation indicates that in the conflict condition, presentation of the rewDS potentiates alcohol seeking, and this is reflected in two behavioral measures, both the punished lever press and the unpunished magazine entries.

In combination, these behaviors can be described as a seeking-taking chain of operant responding (Balleine et al., 1995; Olmstead et al., 2000, 2001). From this perspective, we have punished the seeking response (lever press), but not the taking response (magazine entry). Testing under the punDS completely suppresses both the seeking and taking responses; however, presentation of the punDS in compound with rewDS (conflict) increases alcohol seeking with no effect on the unpunished taking response. These findings indicate the separation of alcohol seeking behaviors and alcohol consumption behaviors, which is interesting to study further in the context of motivational conflict.

It is interesting to note that we also observe a large degree of individual variability in the response to unpredictable punishment in the conflict trials, which is comparable to what we identified previously in the context design (Marchant et al., 2018). The nature of variation in the response to punishment was recently identified to be unrelated to fear learning in a food pellet reinforced task (Jean-Richard-Dit-Bressel et al., 2019). While the variation we observed in this experiment is more pronounced in the conflict trials, the rate of alcohol seeking in conflict positively correlates with the rate in punDS trials (Figure 3C). This correlation implies that there is a common mechanism suppressing alcohol responding in both the punDS and conflict trials, and it is likely that this mechanism is the sensitivity of individual rat to punishment, rather than a shift in probability of shock from 100% in punDS to 50% in conflict.

### Neural Activity Associated With Alcohol Seeking in the Face of Punishment

Overall, the Fos data are surprising in the lack of differences between the punDS and rewDS conditions. This is something which is quite different to typical observations in relapse tests, where the rats that are tested in the alcohol-associated context show higher alcohol seeking, which is typically associated with increased Fos compared to the rats who show lower alcohol seeking in the extinction or punishment context (Hamlin et al., 2007; Marchant et al., 2009, 2014, 2016). One important difference with the tests in this study, however, is they are reinforced sessions and not in extinction which is commonly used in the relapse tests, making direct comparisons difficult because of the potential representation of the outcomes (alcohol or shock) being reflected in the Fos activity. We conducted reinforced tests because we were primarily interested in capturing the approach-avoidance conflict that is present in the conflict trials. In the absence of punishment, the rats in the conflict test condition will immediately extinguish the punishment contingency in the final test, confounding this aim. However, from the Fos data presented here, it is not possible to disentangle activity associated with representation of the DS-associated memories and the response-contingent reinforcement of either alcohol or shock. Finally, in this experiment, we have not made any comparison of Fos expression to a no test control group. In the past, we have made such comparisons (Marchant et al., 2014, 2016), and typically we find in most brain regions that the no test group Fos activity is substantially lower than the tested rats. However, without this comparison in this experiment we are unable to determine the extent to which any lack of differences between the tested groups is because activity is higher than baseline in all rats or is in fact not different from baseline.

With these caveats in mind, we observed a number of significant differences between the test conditions in several brain regions. In the LO cortex, we found that both rewDS and punDS tested rats had higher activity compared to the rats tested under conflict. Orbitofrontal cortex (OFC) activity is associated with many functions particularly related to the adjustment of goal-directed behaviors in response to new contingencies or when the outcomes are not presented (Turner and Parkes, 2020). Given that the final test conditions in this experiment were not associated with any change in contingency, rather expression of prior well-learned associations, it is unlikely that LO activity is specifically related to behavioral output.

During conflict, the rat needs to choose between two competing behavioral responses (lever press or suppress). This is the key difference between the rewDS and punDS trials and is the condition upon which the most motivational conflict should be present. That rats tested in the conflict condition had higher Fos expression in PVT, NAc Shell, and LH suggests that activity in these brain regions may be particularly related to arbitration of motivational conflict, more than alcohol seeking (rewDS) or suppression in the face of certain punishment (punDS). Interestingly these regions are all interconnected, PVT sends dense projections to NAc Shell (Li and Kirouac, 2008; Hamlin et al., 2009) and NAc Shell in turn projects to the LH (Petrovich et al., 2005; Marchant et al., 2009), which itself projects to the PVT (Marchant et al., 2010). It is possible therefore that these regions are particularly important in regulating the conflict imposed by the presentation of the rewDS and punDS Cues in compound. Activity in the NAc Shell has been linked to the suppression of inappropriate responses (Ambroggi et al., 2011; Lafferty et al., 2020), and there is evidence that inhibition of NAc Shell can increase punished food self-administration (Piantadosi et al., 2017). PVT has also been linked to coordinating behavioral output in conflicting conditions (Choi et al., 2019).

Finally, our Fos data raise an interesting possibility that the motivational conflict induced by presenting the rewDS and punDS Cues in compound causes a shift away from cortical processing of behavioral output, to a sub-cortical network involving PVT, NAc Shell, and LH. It will be of interest in future studies to identify whether this activity is related to processing of the conflicting DS, or if the increased activity reflects summation of both the positive and negative outcomes that occur in approach-avoidance conflict. Moreover, it will be of interest to determine if other types of motivational conflict, such as approach-approach conflict (Miller, 1944; McNally, 2021), also recruit selective activity in these sub-cortical brain regions, or if it is specific to approach-avoidance conflict.

## Conclusion

In summary, we have described a novel rodent model of relapse where a single Cue DS associated with alcohol self-administration can promote relapse after punishment-imposed abstinence in the presence of another DS. We then used these DS to show that alcohol self-administration and punishment can be bi-directionally controlled in a within-session task. Presenting the two DS in compound resulted in an intermediate rate of alcohol self-administration, potentially reflecting a state of approach-avoidance motivational conflict. Finally, alcohol seeking under risk of punishment was associated with increased activity in three interconnected brain regions, PVT, NAc Shell, and LH.

## Data Availability Statement

The raw data supporting the conclusion of this article will be made available by the authors, without undue reservation.

## Ethics Statement

This study was approved by The Netherlands Central Committee for Animal Experiments (Centrale Commissie Dierproeven) and the Animal Welfare Body (Instantie voor Dierenwelzijn) of the Vrije Universiteit Amsterdam.

## Author Contributions

All authors listed have made a substantial, direct and intellectual contribution to the work, and approved it for publication.

## Conflict of Interest

The authors declare that the research was conducted in the absence of any commercial or financial relationships that could be construed as a potential conflict of interest.

## Publisher’s Note

All claims expressed in this article are solely those of the authors and do not necessarily represent those of their affiliated organizations, or those of the publisher, the editors and the reviewers. Any product that may be evaluated in this article, or claim that may be made by its manufacturer, is not guaranteed or endorsed by the publisher.

## References

[B1] Ambroggi F. Ghazizadeh A. Nicola S. M. Fields H. L. (2011). Roles of nucleus accumbens core and shell in incentive-cue responding and behavioral inhibition. *J. Neurosci.* 31 6820–6830. 10.1523/JNEUROSCI.6491-10.2011 21543612 PMC3145462

[B2] Balleine B. W. Garner C. González F. Dickinson A. (1995). Motivational control of heterogeneous instrumental chains. *J. Exp. Psychol. Anim. Behav. Process.* 21 203–217. 10.1037/0097-7403.21.3.203

[B3] Bankhead P. Loughrey M. B. Fernandez J. A. Dombrowski Y. McArt D. G. Dunne P. D. (2017). QuPath: open source software for digital pathology image analysis. *Sci. Rep.* 7:16878. 10.1038/s41598-017-17204-5 29203879 PMC5715110

[B4] Blume A. W. Schmaling K. B. Marlatt G. A. (2006). Recent drinking consequences, motivation to change, and changes in alcohol consumption over a three month period. *Addict. Behav.* 31 331–338. 10.1016/j.addbeh.2005.05.014 15979813

[B5] Bolles R. C. Holtz R. Dunn T. Hill W. (1980). Comparisons of stimulus-learning and response learning in a punishment situation. *Learn. Motiv.* 11 78–96. 10.1016/0023-9690(80)90022-3

[B6] Bolles R. C. Uhl C. N. Wolfe M. Chase P. B. (1975). Stimulus learning versus response learning in a discriminated punishment situation. *Learn. Motiv.* 6 439–447. 10.1016/0023-9690(75)90002-8

[B7] Bouton M. E. Maren S. McNally G. P. (2020). Behavioral and neurobiological mechanisms of pavlovian and instrumental extinction learning. *Physiol. Rev.* 101 611–681. 10.1152/physrev.00016.2020 32970967 PMC8428921

[B8] Bouton M. E. Schepers S. T. (2015). Renewal after the punishment of free operant behavior. *J. Exp. Psychol. Anim. Learn. Cogn.* 41 81–90. 10.1037/xan0000051 25706548 PMC4339226

[B9] Cannella N. Economidou D. Kallupi M. Stopponi S. Heilig M. Massi M. (2009). Persistent increase of alcohol-seeking evoked by neuropeptide S: an effect mediated by the hypothalamic hypocretin system. *Neuropsychopharmacology* 34 2125–2134. 10.1038/npp.2009.37 19322167

[B10] Choi E. A. Jean-Richard-Dit-Bressel P. Clifford C. W. G. McNally G. P. (2019). Paraventricular thalamus controls behavior during motivational conflict. *J. Neurosci.* 39 4945–4958. 10.1523/JNEUROSCI.2480-18.2019 30979815 PMC6670259

[B11] Crombag H. S. Badiani A. Maren S. Robinson T. E. (2000). The role of contextual versus discrete drug-associated cues in promoting the induction of psychomotor sensitization to intravenous amphetamine. *Behav. Brain Res.* 116 1–22. 10.1016/s0166-4328(00)00243-6 11090882

[B12] Crombag H. S. Bossert J. M. Koya E. Shaham Y. (2008). Review. Context-induced relapse to drug seeking: a review. *Philos. Trans. R. Soc. Lond. B Biol. Sci.* 363 3233–3243. 10.1098/rstb.2008.0090 18640922 PMC2607323

[B13] Everitt B. J. Belin D. Economidou D. Pelloux Y. Dalley J. W. Robbins T. W. (2008). Review. Neural mechanisms underlying the vulnerability to develop compulsive drug-seeking habits and addiction. *Philos. Trans. R. Soc. Lond. B Biol. Sci.* 363 3125–3135. 10.1098/rstb.2008.0089 18640910 PMC2607322

[B14] Figee M. Vink M. de Geus F. Vulink N. Veltman D. J. Westenberg H. (2011). Dysfunctional reward circuitry in obsessive-compulsive disorder. *Biol. Psychiatry* 69 867–874. 10.1016/j.biopsych.2010.12.003 21272861

[B15] Fraser K. M. Holland P. C. (2019). Occasion setting. *Behav. Neurosci.* 133 145–175. 10.1037/bne0000306 30907616 PMC6447318

[B16] Gray J. A. McNaughton N. (2000). *The Neuropsychology of Anxiety: An Enquiry into the Functions of the Septo-Hippocampal System*, 2nd Edn. Oxford: Oxford University Press.

[B17] Grimm J. W. Hope B. T. Wise R. A. Shaham Y. (2001). Neuroadaptation: incubation of cocaine craving after withdrawal. *Nature* 412 141–142. 11449260 10.1038/35084134 PMC2889613

[B18] Hamlin A. S. Clemens K. J. Choi E. A. McNally G. P. (2009). Paraventricular thalamus mediates context-induced reinstatement (renewal) of extinguished reward seeking. *Eur. J. Neurosci.* 29 802–812. 10.1111/j.1460-9568.2009.06623.x 19200064

[B19] Hamlin A. S. Newby J. McNally G. P. (2007). The neural correlates and role of D1 dopamine receptors in renewal of extinguished alcohol-seeking. *Neuroscience* 146 525–536. 10.1016/j.neuroscience.2007.01.063 17360123

[B20] Hasin D. S. O’Brien C. P. Auriacombe M. Borges G. Bucholz K. Budney A. (2013). DSM-5 criteria for substance use disorders: recommendations and rationale. *Am. J. Psychiatry* 170 834–851. 10.1176/appi.ajp.2013.12060782 23903334 PMC3767415

[B21] Holland P. C. (1992). “Occasion setting in pavlovian conditioning,” in *Psychology of Learning and Motivation*, Vol. Volume ed. Douglas L. M. (Cambridge, MA: Academic Press), 69–125.

[B22] Holland P. C. Bouton M. E. (1999). Hippocampus and context in classical conditioning. *Curr. Opin. Neurobiol.* 9 195–202. 10.1016/s0959-4388(99)80027-0 10322181

[B23] Hopf F. W. Lesscher H. M. (2014). Rodent models for compulsive alcohol intake. *Alchol* 48 253–264. 10.1016/j.alcohol.2014.03.001 24731992 PMC4993047

[B24] Ito R. Lee A. C. H. (2016). The role of the hippocampus in approach-avoidance conflict decision-making: evidence from rodent and human studies. *Behav. Brain Res.* 313 345–357. 10.1016/j.bbr.2016.07.039 27457133

[B25] Jean-Richard-Dit-Bressel P. Ma C. Bradfield L. A. Killcross S. McNally G. P. (2019). Punishment insensitivity emerges from impaired contingency detection, not aversion insensitivity or reward dominance. *Elife* 8:e52765. 10.7554/eLife.52765 31769756 PMC6890457

[B26] Katner S. N. Magalong J. G. Weiss F. (1999). Reinstatement of alcohol-seeking behavior by drug-associated discriminative stimuli after prolonged extinction in the rat. *Neuropsychopharmacology* 20 471–479. 10.1016/S0893-133X(98)00084-0 10192827

[B27] Klingemann H. K. (1991). The motivation for change from problem alcohol and heroin use. *Br. J. Addict.* 86 727–744. 10.1111/j.1360-0443.1991.tb03099.x 1878623

[B28] Lafferty C. K. Yang A. K. Mendoza J. A. Britt J. P. (2020). Nucleus accumbens cell type- and input-specific suppression of unproductive reward seeking. *Cell Rep.* 30 3729–3742.e3. 10.1016/j.celrep.2020.02.095 32187545

[B29] Li S. Kirouac G. J. (2008). Projections from the paraventricular nucleus of the thalamus to the forebrain, with special emphasis on the extended amygdala. *J. Comp. Neurol.* 506 263–287. 10.1002/cne.21502 18022956

[B30] Madangopal R. Ramsey L. A. Weber S. J. Brenner M. B. Lennon V. A. Drake O. R. (2021). Inactivation of the infralimbic cortex decreases discriminative stimulus-controlled relapse to cocaine seeking in rats. *Neuropsychopharmacology* 46 1969–1980. 10.1038/s41386-021-01067-6 34162997 PMC8429767

[B31] Madangopal R. Tunstall B. J. Komer L. E. Weber S. J. Hoots J. K. Lennon V. A. (2019). Discriminative stimuli are sufficient for incubation of cocaine craving. *Elife* 8:e44427. 10.7554/eLife.44427 30801248 PMC6417857

[B32] Marchant N. J. Campbell E. J. Kaganovsky K. (2018). Punishment of alcohol-reinforced responding in alcohol preferring P rats reveals a bimodal population: implications for models of compulsive drug seeking. *Prog. Neuropsychopharmacol. Biol. Psychiatry* 87(Pt A) 68–77. 10.1016/j.pnpbp.2017.07.020 28754407 PMC5785579

[B33] Marchant N. J. Campbell E. J. Pelloux Y. Bossert J. M. Shaham Y. (2019). Context-induced relapse after extinction versus punishment: similarities and differences. *Psychopharmacology (Berl)* 236 439–448. 10.1007/s00213-018-4929-1 29799072 PMC6373446

[B34] Marchant N. J. Campbell E. J. Whitaker L. R. Harvey B. K. Kaganovsky K. Adhikary S. (2016). Role of ventral subiculum in context-induced relapse to alcohol seeking after punishment-imposed abstinence. *J. Neurosci.* 36 3281–3294. 10.1523/JNEUROSCI.4299-15.2016 26985037 PMC4792939

[B35] Marchant N. J. Furlong T. M. McNally G. P. (2010). Medial dorsal hypothalamus mediates the inhibition of reward seeking after extinction. *J. Neurosci.* 30 14102–14115. 20962231 10.1523/JNEUROSCI.4079-10.2010 PMC6634760

[B36] Marchant N. J. Hamlin A. S. McNally G. P. (2009). Lateral hypothalamus is required for context-induced reinstatement of extinguished reward seeking. *J. Neurosci.* 29 1331–1342. 10.1523/JNEUROSCI.5194-08.2009 19193880 PMC6666089

[B37] Marchant N. J. Khuc T. N. Pickens C. L. Bonci A. Shaham Y. (2013). Context-induced relapse to alcohol seeking after punishment in a rat model. *Biol. Psychiatry* 73 256–262. 10.1016/j.biopsych.2012.07.007 22883434 PMC3517691

[B38] Marchant N. J. Rabei R. Kaganovsky K. Caprioli D. Bossert J. M. Bonci A. (2014). A critical role of lateral hypothalamus in context-induced relapse to alcohol seeking after punishment-imposed abstinence. *J. Neurosci.* 34 7447–7457. 10.1523/JNEUROSCI.0256-14.2014 24872550 PMC4035512

[B39] Marinelli P. W. Funk D. Harding S. Li Z. Juzytsch W. Le A. D. (2009). Roles of opioid receptor subtypes in mediating alcohol-seeking induced by discrete cues and context. *Eur. J. Neurosci.* 30 671–678. 10.1111/j.1460-9568.2009.06851.x 19686472 PMC2772149

[B40] McNally G. P. (2021). Motivational competition and the paraventricular thalamus. *Neurosci. Biobehav. Rev.* 125 193–207. 10.1016/j.neubiorev.2021.02.021 33609570

[B41] Milad M. R. Furtak S. C. Greenberg J. L. Keshaviah A. Im J. J. Falkenstein M. J. (2013). Deficits in conditioned fear extinction in obsessive-compulsive disorder and neurobiological changes in the fear circuit. *JAMA Psychiatry* 70 608–618;quiz554. 10.1001/jamapsychiatry.2013.914 23740049

[B42] Miller N. E. (1944). “Experimental studies of conflict,” in *Personality and the Behavior Disorders*, ed. Hunt J. M. (New York, NY: Ronald Press), 431–465.

[B43] Murray M. Nevin J. A. (1967). Some effects of correlation between response-contingent shock and reinforcement. *J. Exp. Anal. Behav.* 10 301–309. 10.1901/jeab.1967.10-301 6056803 PMC1338286

[B44] Naqvi N. H. Gaznick N. Tranel D. Bechara A. (2014). The insula: a critical neural substrate for craving and drug seeking under conflict and risk. *Ann. N. Y. Acad. Sci.* 1316 53–70. 10.1111/nyas.12415 24690001 PMC4114146

[B45] O’Brien C. P. Childress A. R. McLellan A. T. Ehrman R. (1992). Classical conditioning in drug-dependent humans. *Ann. N. Y. Acad. Sci.* 654 400–415. 10.1111/j.1749-6632.1992.tb25984.x 1632593

[B46] Olmstead M. C. Lafond M. V. Everitt B. J. Dickinson A. (2001). Cocaine seeking by rats is a goal-directed action. *Behav. Neurosci.* 115 394–402. 11345964

[B47] Olmstead M. C. Parkinson J. A. Miles F. J. Everitt B. J. Dickinson A. (2000). Cocaine-seeking by rats: regulation, reinforcement and activation. *Psychopharmacology (Berl)* 152 123–131. 10.1007/s002130000498 11057515

[B48] Paxinos G. Watson C. (2005). *The Rat Brain in Stereotaxic Coordinates*, Compact 6th Edition. New York, NY: Academic Press.

[B49] Pennartz C. M. Ito R. Verschure P. F. Battaglia F. P. Robbins T. W. (2011). The hippocampal-striatal axis in learning, prediction and goal-directed behavior. *Trends Neurosci.* 34 548–559. 10.1016/j.tins.2011.08.001 21889806

[B50] Petrovich G. D. Holland P. C. Gallagher M. (2005). Amygdalar and prefrontal pathways to the lateral hypothalamus are activated by a learned cue that stimulates eating. *J. Neurosci.* 25 8295–8302. 10.1523/JNEUROSCI.2480-05.2005 16148237 PMC6725549

[B51] Piantadosi P. T. Yeates D. C. M. Wilkins M. Floresco S. B. (2017). Contributions of basolateral amygdala and nucleus accumbens subregions to mediating motivational conflict during punished reward-seeking. *Neurobiol. Learn. Mem.* 140 92–105. 10.1016/j.nlm.2017.02.017 28242266

[B52] Simms J. A. Steensland P. Medina B. Abernathy K. E. Chandler L. J. Wise R. (2008). Intermittent access to 20% ethanol induces high ethanol consumption in Long-Evans and Wistar rats. *Alcohol Clin. Exp. Res.* 32 1816–1823. 10.1111/j.1530-0277.2008.00753.x 18671810 PMC3151464

[B53] Taha S. A. Fields H. L. (2006). Inhibitions of nucleus accumbens neurons encode a gating signal for reward-directed behavior. *J. Neurosci.* 26 217–222. 10.1523/JNEUROSCI.3227-05.2006 16399690 PMC6674301

[B54] Todd T. P. Vurbic D. Bouton M. E. (2014). Mechanisms of renewal after the extinction of discriminated operant behavior. *J. Exp. Psychol. Anim. Learn. Cogn.* 40 355–368. 10.1037/xan0000021 25545982 PMC4280083

[B55] Turner K. M. Parkes S. L. (2020). Prefrontal regulation of behavioural control: evidence from learning theory and translational approaches in rodents. *Neurosci. Biobehav. Rev.* 118 27–41. 32707346 10.1016/j.neubiorev.2020.07.010

[B56] Wikler A. (1973). Dynamics of drug dependence. Implications of a conditioning theory for research and treatment. *Arch. Gen. Psychiatry* 28 611–616. 10.1001/archpsyc.1973.01750350005001 4700675

[B57] Wise R. A. (1973). Voluntary ethanol intake in rats following exposure to ethanol on various schedules. *Psychopharmacologia* 29 203–210. 10.1007/BF00414034 4702273

